# Management Methods and Duration Induces Changes in Soil Microbial Communities of *Carya cathayensis* var. *dabeishansis* Forests

**DOI:** 10.1002/ece3.72173

**Published:** 2025-09-14

**Authors:** Cheng Huang, Hua Liu, Shu‐Yi‐Dan Zhou, Linyun Mou, Lingjun Cui, Lan Yao, Yuhua Ma, Fasih Ullah Haider, Songling Fu, Xu Li

**Affiliations:** ^1^ Hubei Key Laboratory of Biologic Resources Protection and Utilization Hubei Minzu University Enshi China; ^2^ Anhui Provincial Key Laboratory of Forest Resources and Silviculture Anhui Agricultural University Hefei China; ^3^ National Ecological Science Data Center Guangdong Branch, South China Botanical Garden, Chinese Academy of Sciences Guangzhou China; ^4^ College of Civil and Architecture Engineering Chuzhou University Chuzhou China

**Keywords:** *Carya cathayensis* var. *dabeishansis*, forest management, land degradation, microbial community assembly, microbial co‐occurrence network

## Abstract

Soil microbial communities are involved in and contribute to several processes in soil ecosystems. Nonetheless, how various forest management approaches and their timeframes influence soil microbial community composition and network complexity is poorly understood. Hence, in this study, a time‐series method examined how microbial populations in the soil of *Carya cathayensis* var. *dabeishansis* forests varied across different management practices (no management, extensive management, and intensive management) and over periods of 0, 3, 8, 15, and 20 years. High‐throughput sequencing determined the species composition of soil microbial communities, co‐occurrence network analysis assessed interrelationships between communities, and null model theory elucidated deterministic and stochastic processes governing community assembly. The results indicated that under both treatment methods, soil bacterial diversity indices increased compared to the control during short‐term management (3 years), but subsequently declined with further prolonged management duration. Moreover, soil acid phosphatase activity and total potassium levels primarily shaped the bacterial species in the soil, with *Acidobacteriota* (21.96%–31.45%), *Proteobacteria* (22.82%–31.12%), *Actinobacteria* (6.81%–13.05%), and *Chloroflexi* (6.68%–9.67%) representing the most prevalent bacterial taxa. Interactions between soil bacterial and fungal communities were predominantly cooperative across both management strategies (79.88%–100%). However, the degree of cooperation fluctuated throughout the duration. Stochastic processes, particularly diffusion limitation, played a key role in shaping the assembly of these microbial communities. The diffusion limitation of soil microorganisms was smaller in extensively managed forests than in intensively managed forests. These results highlight the need for balanced forest management strategies, where short‐term intensive practices could help preserve soil microbial diversity and sustain ecosystem functions. Therefore, we strongly recommend adopting an intermittent forest management approach, particularly in intensively managed forests, where it is necessary to allow the ecosystem adequate time for autonomous recovery.

## Graphical abstract

The interspecific relationships between soil bacterial and fungal communities were mainly collaborative in both extensive management and intensive management; Sustained forest management negatively affects soil microbial diversity, composition, and network complexity; Soil microbial community assembly processes diverge under different management methods.

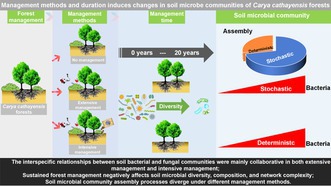

## 1 Introduction

Soil microorganisms drive critical biochemical processes, with their species diversity and community structure playing a pivotal role in sustaining soil ecosystem multifunctionality and resilience (Li, Jiang, et al. 2024; Zhao et al. 2024; Kang et al. 2025; Cabrera‐Hernandez et al. 2025). Research has demonstrated that the diversity and structural intricacy of soil microbial communities are key indicators of ecosystem health and function (Wagg et al. 2019; Zhou et al. 2023; Li, Li, et al. 2024; Li, Wu, et al. 2024). Furthermore, these microbial attributes are highly responsive to variations in forest management practices and when such management is applied (Zheng et al. 2022). Notably, prolonged intensive land use has been linked to declines in soil organic matter, which subsequently alters soil physicochemical conditions and nutrient availability, adversely impacting microbial diversity (Mushinski et al. 2019; Kostin et al. 2021). Fertilizer application is one of the most common management tools in forest management. The application of different fertilizers directly affects the types and pathways of nutrient extraction by soil microbes. Studies have shown that continuous application of nitrogen (N) fertilizers affects the soil NH_4_^+^ and NO_3_^−^ rate and may ultimately increase denitrification (Zheng et al. 2022). Additionally, shallow tillage performed during fertilizer application is a mild soil disturbance that improves aeration, mobilizes organic matter, and generates novel habitats for soil microbial communities (Degrune et al. 2017). Understory vegetation removal and heavy fertilization adopted during forest management will probably lead to soil acidification, reduced soil nutrient effectiveness, and soil aggregate destruction, directly or indirectly altering soil microbial community structure and function (Chen et al. 2019). Long‐term intensive management in subtropical bamboo forests has been found to cause excessive accumulation of soil carbon (C), N, and phosphorus (P), which adversely impacts soil microbial activity and the functioning of nutrient‐cycling enzymes (Yao et al. 2022). Among these factors, N addition‐induced phosphorus limitation emerges as a key driver altering the structure of soil microbial communities (Zheng et al. 2022). The impact of fertilization on soil microbial diversity is not universally negative. In organically managed tea gardens, organic fertilizer application enhances microbial diversity through niche differentiation, promoting copiotrophic groups over oligotrophic groups under resource‐rich conditions (Pu et al. 2025). Additionally, plant species diversity, along with the dynamic interactions between root systems and soil microorganisms during plant development, plays a crucial role in shaping both the composition and functional potential of soil microbial communities (Wang, Tian, et al. 2025). In particular, plant roots and their secretions have made significant contributions to improving soil enzyme activity and soil nutrient release, which can affect the activity of soil microorganisms (Kriiska et al. 2021; Staszel et al. 2022; Staszel‐Szlachta et al. 2024). For instance, Meier et al. (2020) found in *Fagus sylvatica* forests that in highly acidic and nitrogen‐poor soils, an increase in root exudation would reduce fungal activity. For instance, intensive fertilization applied in *Pinus koraiensis* forests to increase pine nut yield alters fine root growth status, reduces bacterial and fungal biomass, and modifies soil microbial community structure (Jia et al. 2025).

The composition and interactions within soil microbial communities are influenced by the physicochemical characteristics of both the soil and the associated plants (Xiao et al. 2023). It is generally believed that a vegetative environment with higher species richness can provide richer resources and spatial heterogeneity for soil microorganisms, which is conducive to increasing soil microbial species diversity and functional diversity (Li et al. 2023). Research indicates that intensive forest harvesting and removal of understory vegetation lead to alterations in vegetation cover and organic inputs, which in turn modify soil organic matter content and reduce the relative abundance and activity of soil bacterial and fungal populations (Mushinski et al. 2019). Studies on hickory (*Carya cathayensis*) understory herbaceous cultivation reveal that prolonged herbaceous growth significantly shifts soil fungal community composition by increasing the prevalence of *Ascomycota*‐fungi that preferentially degrade labile organic compounds while suppressing *Basidiomycota*, which are responsible for breaking down more resistant organic material (Yan et al. 2020). Moreover, the composition and functionality of soil microbial communities are primarily governed by plant root systems, which continuously release root exudates and shed tissues that attract beneficial microbes. These microbes enhance plant growth and mitigate environmental stresses, linking increased plant abundance and coverage to greater soil microbial species and functional diversity (Liu et al. 2019; López‐Angulo et al. 2020). Environmental factors such as soil moisture and temperature also critically influence microbial activity. The influence of forest vegetation management practices on soil microbial community assembly is substantial. For instance, in *Ziziphus jujuba* orchards, straw mulch and orchard grass management increase the connectivity and complexity of soil bacterial‐fungal co‐occurrence networks (Kang et al. 2025). Removing understory vegetation alters surface soil temperature and moisture regimes, triggering the restructuring of microbial communities and affecting bacterial and fungal species composition and functional dynamics (Metze et al. 2024; Zhang et al. 2024).

The formation of soil microbial communities is primarily governed by their assembly processes, with the resulting community structure reflecting how microbes respond to external environmental disturbances. Understanding these assembly patterns is essential for elucidating the links between microbial communities and ecosystem functions (He et al. 2022). It is widely accepted that microbial community assembly is driven by two fundamental ecological mechanisms: deterministic processes, as described by niche theory, and stochastic processes, as outlined in neutral theory (Zhang et al. 2023; Wang et al. 2023). Despite this, limited research has explored how these assembly mechanisms vary under different forest management regimes and durations, leaving the responses of soil microbial community structure and ecological function to forest management largely unresolved. For instance, during forest management, how stochastic and deterministic processes governing microbial community assembly shift remains unclear. Furthermore, whether these shifts are primarily driven by management methods or management duration lacks definitive conclusions in the current literature.

*Carya cathayensis* var. *dabeishansis* (*C. cathayensis*) is an endemic genetic resource native to China's Dabie Mountains, where extensive natural forests were first identified in the 1970s. Due to the high nutritional value and excellent flavor of its nuts, cultivation of this species has expanded significantly over the past 20 years. During this period, large‐scale harvesting and management of local *C. cathayensis* forests have been undertaken to maximize fruit production. However, previous research indicates that prolonged forest management can suppress soil microbial activity, alter microbial community composition, affect the secretion of extracellular enzymes, and ultimately impair the ecological functions of soil microbial communities (Chen et al. 2022; Zheng et al. 2022; Zhang et al. 2023). This study aimed to elucidate how forest management practices influence soil microbial communities' assembly and ecological roles over time by comparing microbial community characteristics and functions across different management regimes and durations in *C. cathayensis* forests. It was hypothesized that (1) soil bacterial and fungal diversity would initially rise and then decline with increasing management duration; (2) dominant microbial functional roles would vary according to management type; and (3) intensive management would enhance deterministic assembly processes of soil microbes driven by nutrient availability. The outcomes of this research contribute to a deeper understanding of soil microbial ecological responses to forest management and provide valuable insights for promoting sustainable management strategies for *C. cathayensis* forests.

## 2 Materials and Methods

### 2.1 Study Area

The study sites were situated in the Dabie Mountain Forest Area, Tiantangzhai Town (115°50′28″E, 31°15′32″N), and Guanmiao Township (115°23′13″E, 31°27′57″N) in LuAn City, Anhui Province, China. This region experiences a humid subtropical monsoon climate with an annual average precipitation of 1300 mm (average summer and winter temperature is 26°C and 2°C). The soil type was primarily Humic Cambisols soil (thickness ranges from 30 cm to 100 cm, pH ranges from 4.5 to 6.5). More than 90% of the forest area of the tree layer vegetation is dominated by *C. cathayensis*. Still, there were also small amounts of *Cunninghamia lanceolata* , *Fortunearia sinensis*, and *Liquidambar formosana* (Huang, Fu, Ma, et al. 2023; Huang, Fu, Tong, et al. 2023). The understorey vegetation consists mainly of *Duchesnea indica* , *Erigeron annuus* , and *Aster lautureanus*.

### 2.2 Sampling Design

This study has focused on *C. cathayensis* forests. To ensure that different management methods and their duration were the driving factors behind changes in the stand characteristics of the experimental plots, the selected sites had to meet the following criteria (Hurlbert 1984): (1) similar site conditions, including parent rock, topographical position, slope, aspect, stand density, and canopy closure; (2) the exact stand originsSpecifically, specifically naturally regenerated forests following harvesting in the 1980s; (3) comparable stand ages (35–40 years) and similar original stand compositions; (4) uniform management by a collective organization. Based on the definitions and classification criteria established by preceding studies on forest management methods for *Phyllostachys edulis* forests (Ni et al. 2021) and *Castanea mollissima* forests (Li et al. 2014), the management methods for *C. cathayensis* forests were categorized into three types: unmanaged (CK), extensive management (EM), and intensive management (IM).

Based on census data provided by the local forestry station and visits to the managing units to determine the actual onset times of management activities, the following time intervals were selected for the management duration: 0 years (CK), 3 years (IM3), 8 years (IM8), 15 years (IM15), and 20 years (IM20) (Table S1). The measures implemented under different forest management practices were as follows: CK forests: The original stand composition was preserved, with only the mature seeds of *C. cathayensis* being collected in the autumn and no other anthropogenic disturbances occurring in the area. EM forests: An extensive and straightforward approach to forest management was applied, including removing almost all tree and shrub species except for *C. cathayensis*. Additionally, the understory vegetation was cleared once a year, before the nut harvest (in late August), with no fertilization applied. IM forests: This management practice also involved the removal of almost all tree and shrub species in the forest, except for *C. cathayensis*. A compound fertilizer (N: P_2_O_5_: K_2_O = 13:5:7) (375 kg ha^−1^) was applied in May of each year. Further, the understory vegetation was cleared twice annually (July and late August). A commercial organic fertilizer (45% organic matter content) (1500 kg ha^−1^) was applied in late September.

Initially, the historical forest survey data from the Forestry Station were employed to identify all stands that met the experimental design criteria. Subsequently, a target stand was randomly selected from these preliminarily identified stands. Further validation entailed field visits and on‐site assessments to ensure that the selected stands demonstrated comparable site conditions, parent rock characteristics, soil texture similarities, consistent management practices, and identical initiation times for management activities. Utilizing this methodology, five plots (20 m × 20 m; 400 m^2^) were randomly established to represent each distinct forest management strategy and temporal category of *C. cathayensis* forests (CK, EM3, EM8, EM15, EM20; IM3, IM8, IM15, IM20), which resulted in a total of 45 plots (five plots × nine stands). According to the actual terrain characteristics of the sample sites, we ensured that the edge distance between each quadrangle was > 200 m. For each plot, a 20 m × 20 m section was randomly established to represent the forest stand, and all plots were set on the mountain's northern slope (20°–30°) at > 100 m from the road and farmland. To reduce spatial auto‐correlations, the distance between each forest area was > 300 m, where each spanned an area of over 2 ha.

### 2.3 Sample Collection

In July 2022, soil samples were collected from three depth intervals (0–10 cm, 10–20 cm, and 20–30 cm) within each *C. cathayensis* forest stand, with five random replicates per stand. Five soil cores were extracted using a 3.5 cm diameter auger for each depth and combined to create a composite sample. 135 soil samples were obtained from nine stands (three depths × five plots × nine stands). After removing fine roots and stones, samples were sealed and transported to the laboratory under refrigeration at 4°C for further analysis.

### 2.4 Laboratory Analysis

Fresh soil samples were used to detect total soil protease (Pro), sucrase (SC), acid phosphatase (ACP), β‐1,4‐glucosidase (BG), microbial biomass carbon (MBC), microbial biomass nitrogen (MBN), and microbial biomass phosphorus (MBP). Soil bulk density (BD) was measured using the ring knife method. Total soil porosity (SP) was determined by the specific gravity method (Yang et al. 2021). After air drying, soil urease (UE), pH, conductivity (EC), organic carbon (SOC), total nitrogen (TN), total phosphorus (TP), total potassium (TK), alkali hydrolyzable nitrogen (AN), available phosphorus (AP), and available potassium (AK) were measured through a 100‐mesh sieve. The soil pH was measured using a pH meter (Mettler Toledo, FE28‐Standard, Switzerland) in a 1:2.5 soil‐water leaching solution. The EC values were determined with a conductivity meter (Mettler Toledo, FE38‐Standard, Switzerland) at 25°C in a 1:5 soil‐water leachate (Li et al. 2025). Soil TN was determined using a Kjeldahl nitrogen analyzer, SOC content by the potassium dichromate oxidation‐external heating method, and MBC, MBN, and MBP by chloroform fumigation extraction (Zhao et al. 2024). TP and AP were determined using molybdenum‐antimony anti‐spectrophotometry, AN was detected by the alkaline hydrolysis diffusion method, and TK and AK were determined using a flame photometer (SHERWOOD, M410, UK). T‐Pro, S‐SC, S‐ACP, S‐β‐GC, and S‐UE were detected using kits (ADS‐W‐D008, ADS‐W‐TR007‐96, ADS‐W‐TR008, ADS‐W‐TR003‐96, ADS‐W‐TR001‐96), and all kits were provided by Jiangsu (China) Aidisheng Biotechnology Co. Ltd.

### 2.5 DNA Extraction, Amplification and Sequencing

Approximately 0.5 g of homogenized fresh soil was used for total DNA extraction, employing the CTAB protocol. DNA quality was assessed by agarose gel electrophoresis, and quantification was performed using a UV spectrophotometer (Zhu et al. 2024; Ni et al. 2025). For bacterial community analysis, the V3–V4 hypervariable region of the 16S rRNA gene was amplified using primers 341F (5′‐CCTACGGGGNGGCWGCAG‐3′) and 805R (5′‐GACTACHVGGGTATCTAATCC‐3′). Fungal ITS2 regions were targeted with primers ITS1FI2 (5′‐GTGARTCATCGAATCTTTG‐3′) and ITS2 (5′‐TCCTCCGCTTATTGATATATGC‐3′) (Zhou et al. 2023; Wang, Lin, et al. 2024; Wang, Liu, et al. 2024; Wang, Xu, et al. 2024). PCR reactions were conducted using Phusion Hot Start Flex 2X Master Mix in a 50 μL volume comprising 12.5 μL master mix, 2.5 μL each of forward and reverse primers, 2.5 μL template DNA (~50 ng), and nuclease‐free water. Thermal cycling included an initial denaturation at 98°C for 30 s, followed by 35 cycles of 98°C for 10 s, annealing at 54°C for 30 s, and extension at 72°C for 45 s, with a final extension at 72°C for 10 min. Amplification success was verified on 2% agarose gels. PCR products were purified using magnetic beads (Beckman Coulter Genomics) and quantified with a Qubit fluorometer (Invitrogen). Library quality was evaluated using an Agilent 2100 Bioanalyzer and quantified with an Illumina library quantification kit (Kapa Biosciences). Sequence quality control and denoising were performed with QIIME2's DADA2 plugin. High‐quality sequences were clustered into Amplicon Sequence Variants (ASVs) at 99% similarity using UPARSE. Taxonomic assignment of bacterial and fungal ASVs was conducted using the SILVA and RDP databases, respectively, resulting in 69,522 bacterial and 11,735 fungal ASVs.

### 2.6 Data Analysis

The current study employed co‐occurrence network analysis to comprehensively evaluate the complex interrelationships among soil microbial taxa under varying forest management approaches to identify potential ecological interactions and community organization patterns. Correlation matrices were systematically generated by calculating all pairwise Spearman correlations between microbial taxa at the genus level, with statistically significant relationships (*p <* 0.01 after Benjamini‐Hochberg correction) retained for network construction. These analyses were implemented using the *Hmisc* package for correlation calculations and the *igraph* package for network manipulation in R version 4.1.3. For network visualization and topological analysis, Gephi 0.9.2 software was utilized, applying the Fruchterman‐Reingold force‐directed layout algorithm with parameters optimized through multiple iterations to achieve optimal node distribution and cluster separation. This algorithm treats edges as springs that attract connected nodes while repelling all node pairs, resulting in a spatial arrangement that effectively reveals community modules and keystone species. Network topological properties, including connectivity, average path length, modularity, and node centrality measures, were quantitatively assessed to characterize microbial communities' structural complexity and stability across different management regimes.

This study implemented a robust null modeling framework incorporating both phylogenetic and taxonomic approaches to disentangle the relative contributions of deterministic versus stochastic processes in shaping microbial community structure. Phylogenetic community turnover was evaluated by calculating the β‐nearest taxon index (βNTI), which measures the standardized effect size of mean phylogenetic distance between communities. Simultaneously, taxonomic β‐diversity was assessed using the Raup‐Crick metric (RC‐Bray) based on Bray‐Curtis dissimilarities, providing complementary insights into assembly mechanisms. Following established protocols (Zhang et al. 2023), we generated null distributions through 999 randomizations of the phylogenetic tree and community matrices, then computed βNTI and RC‐Bray as the standard deviations between observed and null expected β‐diversity values. Communities were classified as dominated by deterministic processes when |βNTI| > 2, with βNTI > 2 indicating heterogeneous selection (environmental filtering promoting phylogenetic divergence) and βNTI < −2 reflecting homogeneous selection (environmental homogenization driving convergence). Stochastic processes, including dispersal limitation, homogenizing dispersal, and ecological drift, were inferred when |βNTI| ≤ 2, with additional interpretation from RC‐Bray values to distinguish among these stochastic mechanisms.

Additionally, this study employed a multifaceted approach to characterizing microbial α‐diversity, incorporating four complementary metrics: (1) ASV richness as a direct count of observed taxa; (2) Simpson's diversity index to quantify dominance patterns; (3) Shannon‐Wiener index to assess species evenness and richness; and (4) Chao1 estimator to predict total species richness including undetected taxa. Differences in diversity measures among management treatments were evaluated using two‐tailed Student's *t*‐tests for normally distributed data or Wilcoxon rank‐sum tests for non‐normal distributions, with significance thresholds set at *p <* 0.05 after verifying assumptions of homogeneity of variance through Levene's tests. To visualize and test compositional differences among microbial communities, we performed principal coordinates analysis (PCoA) based on Bray–Curtis dissimilarity matrices, followed by permutational multivariate analysis of variance (PerMANOVA) with 999 permutations implemented through the *adonis2* function in the *vegan* package. This multivariate framework allowed us to partition variance components attributable to management type, duration, and their interaction while accounting for spatial autocorrelation.

This research conducted a series of integrative analyses to identify key environmental drivers of microbial community structure. First, Pearson correlation analysis and Mantel test were computed between relative abundances of dominant bacterial and fungal phyla and measured soil physicochemical properties. Redundancy analysis (RDA) was performed with forward selection and Monte Carlo permutation tests (999 permutations) using Canoco 5 software to identify the minimal set of environmental variables that best explained community variation. Structural equation modeling (AMOS 24.0) was employed to evaluate hypothetical causal pathways linking forest management practices, soil characteristics, and microbial community attributes, with model fit assessed through *χ* ^2^ tests, comparative fit index (CFI), and root mean square error of approximation (RMSEA). Annotating ASVs against the FAPROTAX database conducted functional profiling of bacterial communities, while fungal functional guilds were predicted using the FUNGuild database accessed through the Micromeritics Biotechnology Cloud Platform (https://www.bioincloud.tech/standalone‐task‐ui/funguild). All statistical analyses and visualizations were implemented in R 4.1.3 using the *ggplot2* package for graphical output, with supplementary null modeling analyses performed on the Tutu Cloud Platform (http://www.cloudtutu.com/#/index) to validate this study's findings.

## 3 Results

### 3.1 Influence of Forest Management Methods and Duration on Species Composition and Diversity of Soil Microbial

The IM3 treatment exhibited the highest values for bacterial community metrics, including ASV richness, Simpson diversity, Shannon‐Wiener index, and Chao 1 richness, with EM3 ranking second. A consistent decline in bacterial diversity indices was observed as management duration increased. By EM15 and EM20, bacterial diversity stabilized across soil layers, though ASV counts and Simpson indices remained significantly reduced compared to unmanaged controls (CK; *p <* 0.05). Under IM, diversity continued to diminish over time. Notably, bacterial diversity in the surface soil layer (0–10 cm) varied markedly among stands (*p <* 0.05), with these differences attenuating in deeper layers (Figure S1).

**FIGURE 1:**
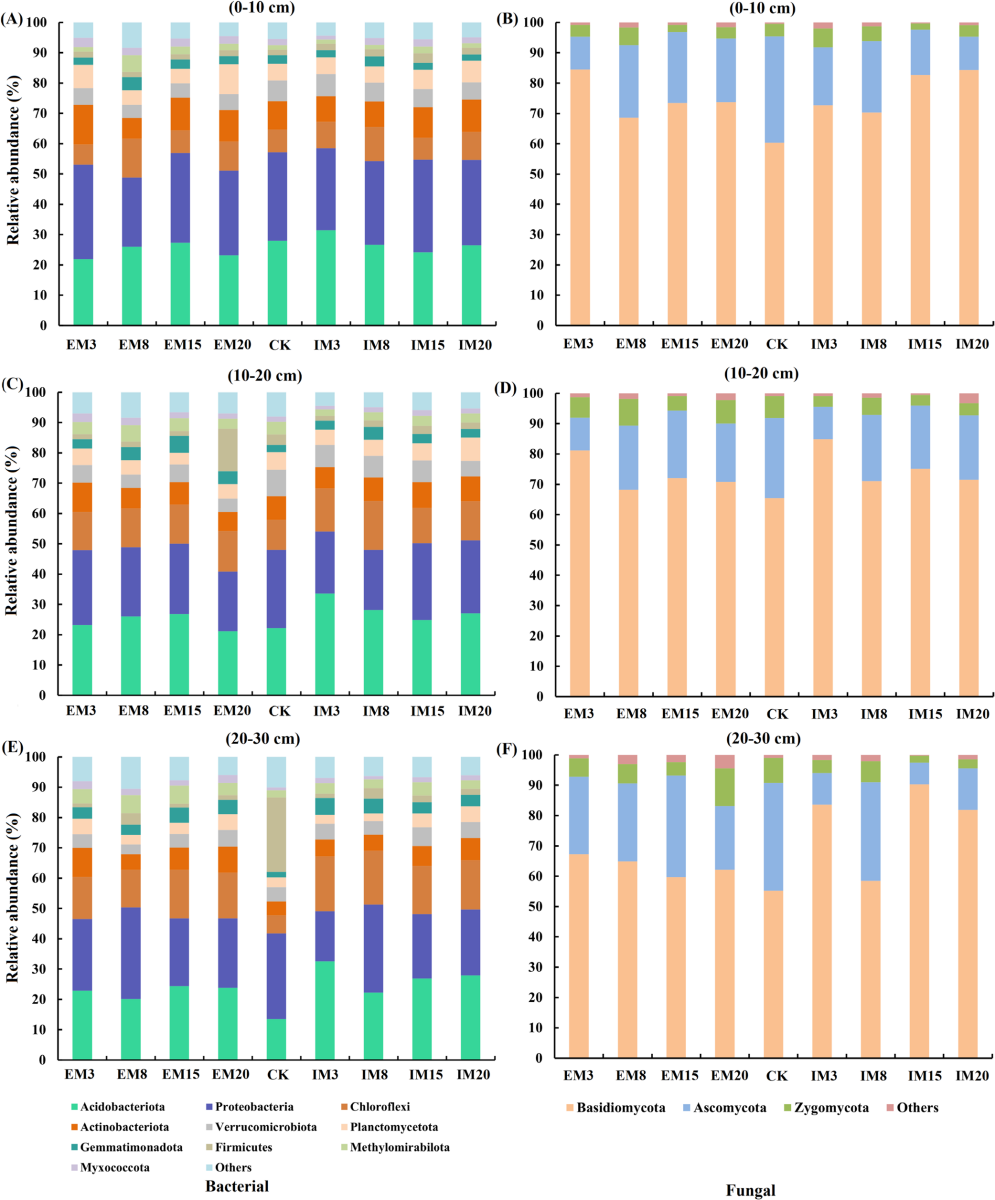
Changes in relative abundance of bacterial and fungal community species (phylum level) by soil depth under different management methods and duration. (A, B) 0–10 results for bacteria and fungi; (C, D) 10–20 results for bacteria and fungi; (E, F) 20–30 results for bacteria and fungi.

**FIGURE 2:**
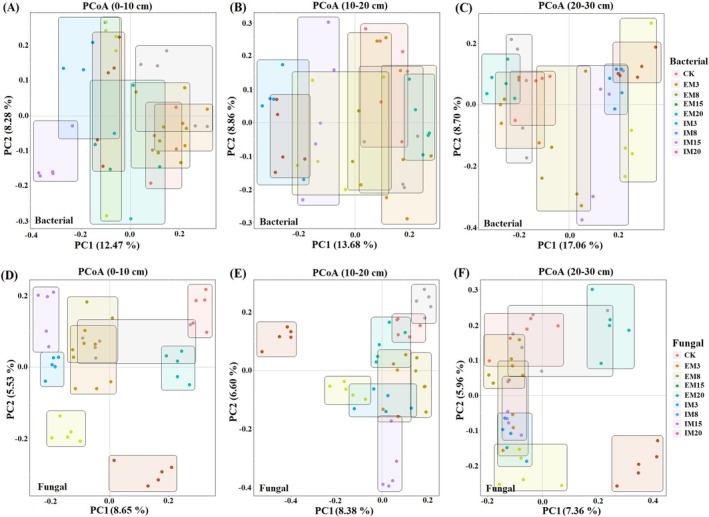
Principal component analysis of species composition based on Bray‐Curtis distances bacterial (A–C) and fungal (D–F) ASV levels.

Fungal communities displayed analogous trends, with the most pronounced diversity shifts occurring in the 0–10 cm layer. Both management approaches (IM, EM) triggered significant reductions in fungal diversity indices (*p <* 0.05). After 15 years, fungal diversity reached a stable but diminished state relative to CK (*p <* 0.05). Bacterial communities were dominated by *Acidobacteriota*, *Proteobacteria*, and *Chloroflexi*, while fungal assemblages were primarily composed of *Basidiomycota*, *Ascomycota*, and *Zygomycota* (Figure 1). IM favored *Ascomycota* over EM at equivalent time points. Conversely, *Zygomycota* abundance increased in all IM soil layers compared to CK. Reductions in *Ascomycota* under IM ranged from 8.81% to 79.96% (0–10 cm), 17.40%–59.58% (10–20 cm), and 32.86%–68.82% (20–30 cm), while EM showed declines of 6.03%–40.98%, 16.13%–59.17%, and 31.83%–69.21%, respectively. PCoA analysis revealed minimal divergence in bacterial composition across management durations within the same treatment, suggesting temporal stability in community structure (Figure 2). However, both IM and EM drove significant compositional restructuring over time. PerMANOVA confirmed distinct bacterial communities in managed stands (0–20 cm layers) versus CK (*p <* 0.05; Table S2). Fungal communities exhibited progressive homogenization after 15 years of management, with diminishing inter‐treatment differences (Table S3).

### 3.2 Influence of Soil Factors on the Characterization of Soil Microbial Communities

**FIGURE 3:**
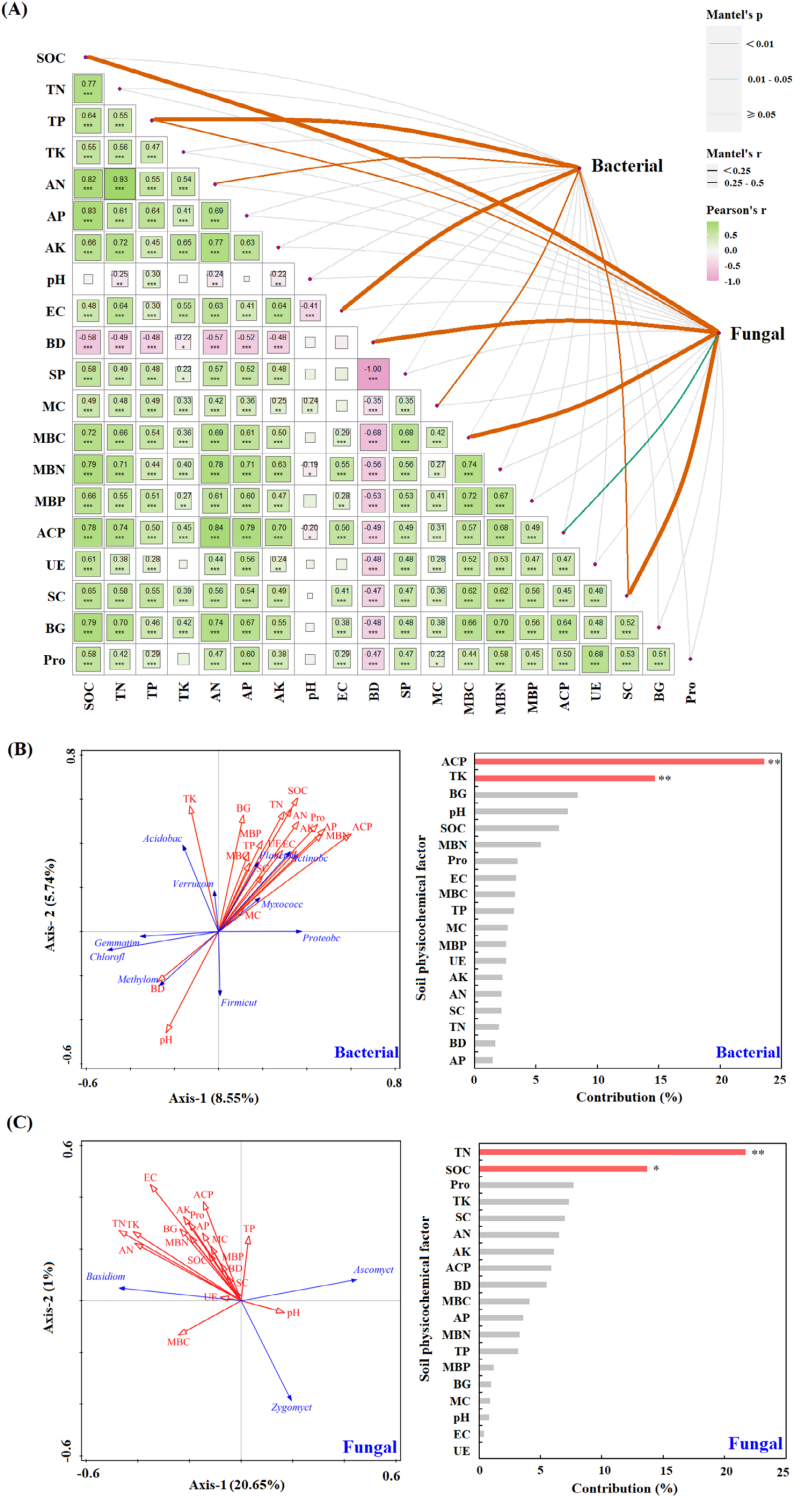
Effects of soil environmental factors on soil microbial diversity and community composition. Side colors indicate their statistical significance based on 9999 permutations, while widths indicate the Mantel test for the corresponding distance correlation (A). Using dominant flora as explanatory variables, key environmental factors influencing soil microbial composition were identified by redundancy analysis and Monde Carlo test (B, C). ***, *p* < 0.001; **, *p* < 0.01; *, *p* < 0.05.

The Mantel's test results showed that the soil TP was significantly and positively correlated with bacterial biodiversity (*p* < 0.01), and the SOC content was significantly and positively correlated with fungal biodiversity (*p* < 0.01). The RDA results showed that the cumulative explanation of the first two axes of soil factors for the changes in the dominant flora of the top 10 relative abundances of soil bacteria was 14.29% (Figure 3B). The cumulative explanation of the first two axes for the changes in the dominant population at the level of soil fungal phyla was 21.65% (Figure 3C). The Monte Carlo test showed that ACP activity and TK were the main factors influencing the composition of the bacterial dominant flora (*p* < 0.01), followed by BG activity, pH, and SOC. TN and SOC significantly affected fungal dominant flora (*p <* 0.05), followed by Pro activity, TK, and SC activity.

**FIGURE 4:**
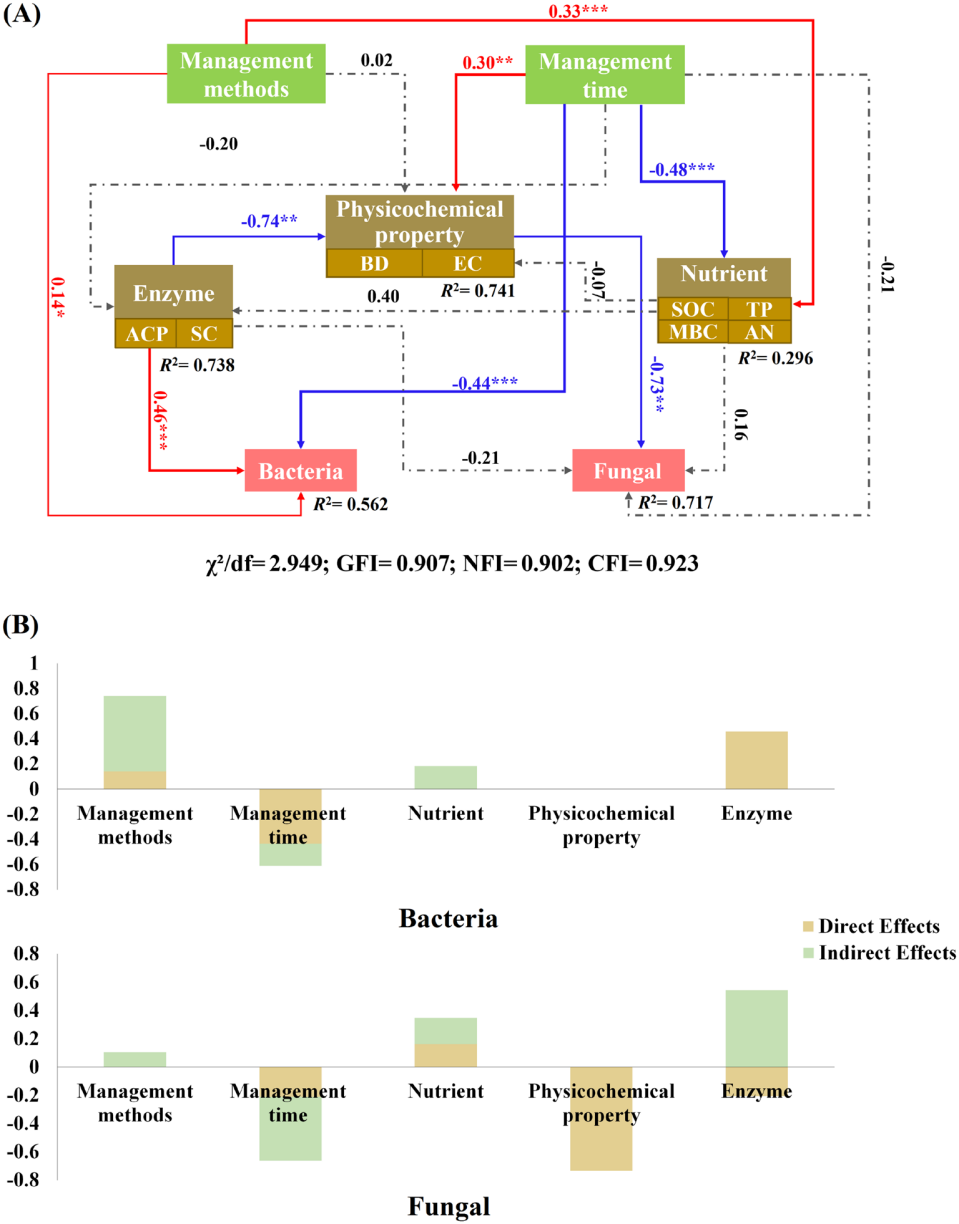
Structural equation modeling (A) of soil microbial community biodiversity driven by forest management methods and duration. Direct and indirect effects of variables (B), solid red lines indicate significant positive effects, solid blue lines indicate significant negative effects, and dashed gray lines indicate non‐significant effects, whether positive or negative. ***, *p* < 0.001; **, *p* < 0.01; *, *p* < 0.05.

The SEM demonstrated acceptable model fit: *χ* ^2^/df = 2.949 (acceptable threshold < 3), with GFI, NFI, and CFI all exceeding the 0.90 benchmark (Figure 4A). Forest management methods, management time, soil extracellular enzyme activity, soil physical and chemical properties, and soil nutrients all directly or indirectly affect the biodiversity of bacterial and fungal communities. The SEM model showed that management methods (*p* < 0.05) and soil extracellular enzyme activity (*p* < 0.001) had substantial and direct adverse effects on bacterial community biodiversity (Figure 4B). Soil physical and chemical properties significantly affected fungal community biodiversity (*p* < 0.01). Still, the direct and indirect effects of management mode and management time on fungal community biodiversity were limited.

### 3.3 Soil Microbial Community Functional Changes, Co‐Occurrence Networks and Assembly Processes

There were no significant differences in the functional characteristics of soil microbial in each stand under different management practices and operating times, and the relative abundance of bacterial taxa with chemoenergetic heterotrophic and aerobic heterotrophic functions was the highest in all stands. The relative abundance of fermentation functional groups was higher in CK stands, while the relative abundance of functional groups for cellulose hydrolysis and N fixation was higher in IM and EM stands (Figure S3). The main fungal dominant functional groups in CK stands were ectomycorrhizal fungi, endophytes, apoplastic saprophytes, and soil saprophytes. The main fungal dominant functional groups in EM forests were ectomycorrhizal fungi, undefined saprophytes, root‐associated fungi, wood‐rotting fungi, and soil‐rotting fungi. IM forests' main fungal dominant functional groups were ectomycorrhizal fungi and wood‐rotting fungi (Figure S3).

**FIGURE 5:**
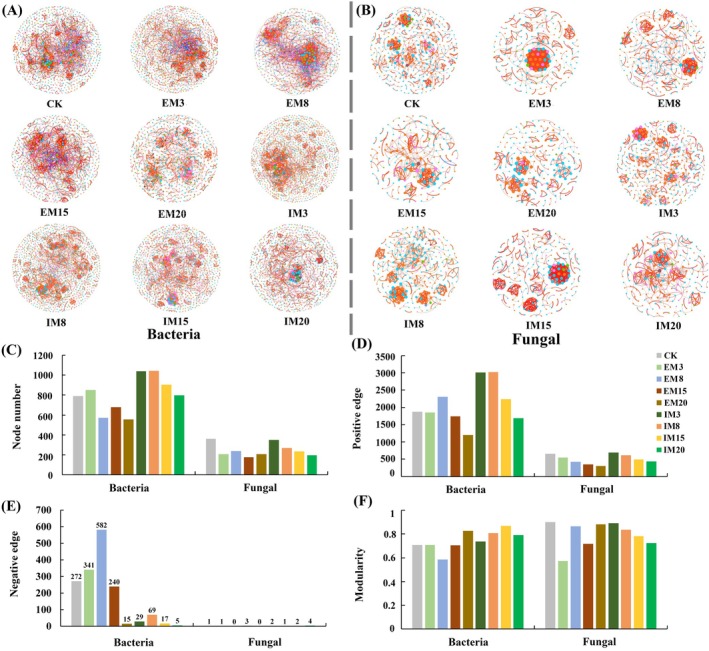
Effect of different forest management methods and duration. Co‐occurrence network of bacterial community (A) and fungal community (B) at the level of ASVs. Nodes of the same species are represented by the same color, and node size corresponds to the number of connections (degrees). Red and green edges indicate positive and negative interactions between nodes. Topological features of the microbial co‐occurrence network after adding different fertilizers (C–F).

Soil bacterial and fungal communities showed different patterns of symbiosis under both IM and EM forests, and the number of nodes and edges in the network structure of bacterial and fungal communities gradually decreased with the increase of management time under both IM and EM forests (Figure 5). The number of positive correlation edges between soil microbial species was higher than the number of negative correlation edges in all stands, indicating that the relationship between species was dominated by positive collaboration. Still, the collaborative effect decreased with the increase in management time. The network structure complexity of bacterial and fungal communities was higher in IM than in EM forests under the same management time. Continuous management activities reduced the interconnections between soil microbial community species. Compared to CK, the number of edges of EM20 and IM20 bacterial communities decreased by 43.21% and 20.95%, respectively. Compared to CK, the number of community edges decreased by 53.31% and 34.04% for EM20 and IM20 fungal communities, respectively.

**FIGURE 6:**
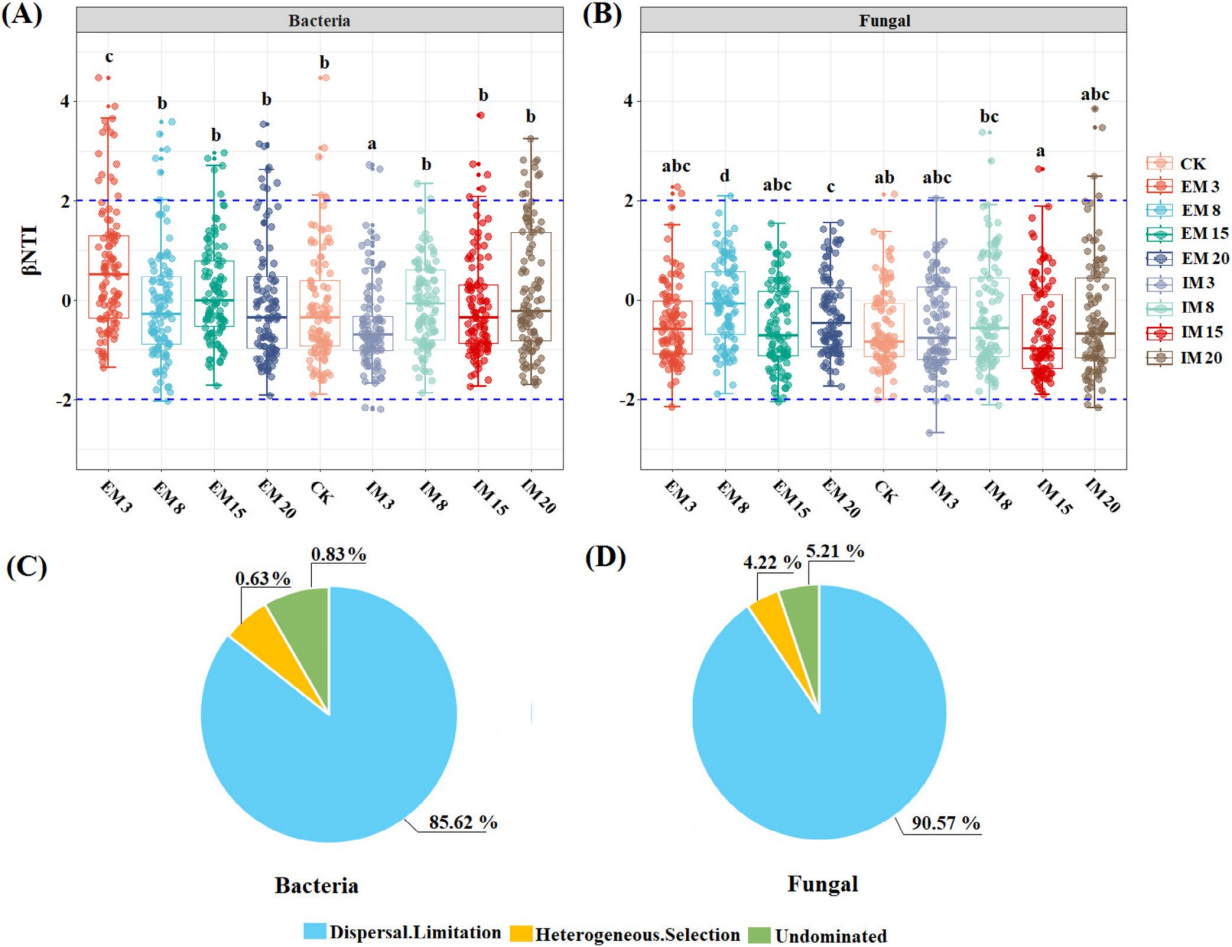
Distribution of βNTI of bacterial (A) and fungal (B) among treatments. The assembly of bacterial (C) and fungal (D) communities is influenced by the percentages of random processes (Dispersal limitation) and deterministic processes (Heterogeneous selection). The LSD multiple testing significance level was *p* < 0.05. Different lowercase letters indicate significant differences between stands.

Deterministic community assembly (|βNTI| > 2) and stochastic assembly (|βNTI| < 2) of soil bacteria and fungi were significantly different (*p* < 0.05) under different management methods and management times (Figure 6). Both soil bacterial and fungal community assembly processes were dominated by stochastic assembly. Still, the trends of deterministic and stochastic assembly processes of soil bacterial communities under IM and EM forests differed with the increased management time. The deterministic assembly of soil bacterial communities in EM forests gradually decreased with the increase in management time, and the stochastic assembly gradually increased, while the deterministic assembly of soil bacterial communities in IM forests gradually increased with the increase in management time, and the stochastic assembly decreased. The contribution of diffusion limitation to bacterial and fungal communities during stochastic assembly was high, 85.62% and 90.57%, respectively.

## 4 Discussion

### 4.1 Effects of Forest Management on Soil Microbial Species Diversity

The management methods and time significantly affected the species diversity of soil microbes in *C. cathayensis* forests. Compared with CK, the number of ASVs of soil bacterial community species, Simpson's diversity index, Shannon‐Wiener's diversity index, and Chao 1 richness index increased. Then, they decreased with the increase of management time, which verified the conjecture of our Hypothesis 1 (Bai et al. 2023; Wu et al. 2019). This may be due to the input of large amounts of plant residues and apoplasts at the beginning of the management, increased substrate heterogeneity, and reduced nutrient competition among soil microbial communities, allowing more soil microorganisms to coexist (Li, Jiang, et al. 2024; Li, Liu, et al. 2024). With the increase in management time, the understory vegetation decreased, the SOM content decreased, the soil microbial activity decreased, and the dominant flora gradually alternated from eutrophic to oligotrophic bacteria (Canini et al. 2019; Xue et al. 2020). Secondly, early anthropogenic disturbances altered the forest stand environment, such as increasing understory light and changing soil permeability, which allowed more microbes with different nutritional modes to migrate into the community (Liu et al. 2023). With the increase in management time, some soil microorganisms were gradually eliminated due to the differences in their adaptability to the environment, and therefore, the species diversity decreased (Ma et al. 2021). In addition, the activities of most soil microbes were related to soil moisture, pH, and the content of limiting nutrient elements. From the physicochemical properties of the forest soil, the SOC and AP content decreased significantly with the increase of management time, so soil pH decreased (Table S4–S6). Plant community composition and species diversity can affect the composition and species diversity of soil microbial communities by altering root secretions, plant apoplasts, and vegetation clearing, leading to the loss of plant diversity, which indirectly alters the structure and composition of soil microbial communities (Cai et al. 2018; Li et al. 2022). Studies indicate that reduced root systems and root exudates significantly impair soil C‐ and N‐cycle enzyme activities (Staszel et al. 2022). This constrains soil nutrient availability, consequently altering microbial community structure and resource acquisition pathways (Kriiska et al. 2021; Staszel‐Szlachta et al. 2024).

### 4.2 Effects of Forest Management on the Composition of Soil Microbial Species

The relative abundance of species in the phylum *Actinomycetes* and *Puccinomycetes* was higher in *C. cathayensis* forests, and the relative abundance of the phylum *Actinomycetes* was higher in both CK and EM forests than in IM forests. The abundance of *Actinomycetes* was related to the health of the Hickory tree because *Actinomycetes* could colonize the plant root and produce antibiotics to inhibit the growth of plant pathogens and improve plant resistance to pathogen infestation (Bai et al. 2023). They produced antibiotics to inhibit the growth of plant pathogens, which can increase plant resistance to pathogen infestation. It was suggested that the risk of disease in *C. cathayensis* forests may be lower under CK and EM compared to IM forests. The RDA results showed that ACP and TK were the main factors affecting the bacterial community composition, which might be because the relative content of K and P in forest soils was lower than the soil N content, especially in IM forests where the elemental composition of fertilizers applied was N:P_2_O_5_:K_2_O = 13:5:7, leading to the possibility that the relative limitation of K and P might restrict the soil bacterial community. Some studies have confirmed that high N addition will limit soil P (Spohn et al. 2016; Zeng et al. 2021). Previously, in tropical forests, K was confirmed to limit soil microbial activity, but in subtropical regions, there was currently no evidence that elemental K has a limiting effect on soil microbial activity, so further validation is needed (Mori et al. 2018).

The dominant phyla of the soil fungal community in *C. cathayensis* forests were more concentrated, with *Stramonium*, *Ascomycetes*, and *Combretum* occupying a total of 98.53% of the total relative abundance of fungal microorganisms, of which *Stramonium* occupied absolute dominance. The genera ranked in the top ten of the relative abundance of the fungi belonged to *Stramonium*, except for *Porphyromonas*. *Ascomycetes* and *Aspergillus* decompose apoplastic litter material and SOM (Zhao et al. 2021). Still, the trend of change in EM forests and IM forests is different with the increased management time. The relative abundance of *Aspergillus* in EM forests increases with the increase of management time, but the trend of change in IM forests was the opposite, which may be associated with the increase in the availability of soil nutrients and vegetation cover (Feng et al. 2018; Li et al. 2022). A large amount of plant residues in the early stage of management increased soil organic matter content, and *Ascomycetes*, which utilize easily degradable organic matter, were able to colonize the soil rapidly. With the increase in management time, the EM forests were still able to maintain relatively high vegetation cover and soil organic carbon. In contrast, the IM forests saw a drastic decrease in vegetation cover, coupled with the accumulation of undecomposable components of the soil from the long‐term application of chemical fertilizers, which created a new opportunity for the *Ascomycetes*, which are adept at utilizing undecomposable organic matter, to colonize the soil. This has created conditions for colonizing *Stachybotrys*, which was good at utilizing undecomposable organic matter (Yan et al. 2020; Zhang et al. 2023).

### 4.3 Effects of Forest Management on Functional Properties and Community Assembly of Soil Microorganisms

From the annotated results of ecological functions of soil bacterial and fungal communities, the main functional groups of soil bacterial communities were the same under different management methods and management times. This was not consistent with our Hypothesis 2. The concept of functional redundancy in microbial communities posits that biodiversity loss may not substantially impair ecological functioning. As Pu et al. (2025) described, microbial diversity and functional potential are asynchronous. This redundancy explains why reduced diversity in intensively managed forests may not impair processes like organic matter decomposition. Although the fermentation functional group had higher abundance in CK stands, and the functional groups of cellulose hydrolysis and N fixation had higher relative abundance in IM and EM forests, none of the differences were significant. Differences in substrate composition may be a key factor contributing to the differences in the abundance of the main functional groups of bacteria, as CK stands are virtually undisturbed and have a high SOM content, thus requiring more bacteria with fermentative decomposition roles to act in substrate decomposition (Ritter et al. 2021). Compared with CK, the higher abundance of wood‐rotting bacteria in IM forests and EM forests, especially in IM forests, may cause the residues left behind by high‐intensity understory cutting, irrigation, and weeding to create conditions for wood‐rotting bacteria to colonize the soil (Yan et al. 2018).

Constructing soil microbial ecological networks and clarifying the process of soil microbial community assembly was important for determining the ecological functions of soil microbes and deciphering microbial symbiosis patterns (Zhang et al. 2023). The interspecific relationships of soil bacterial and fungal species in this study were mainly dominated by positive synergism, consistent with the results of previous related studies (Sun et al. 2024). Regarding microbial ecological network and topology, the correlation between soil microbial species significantly decreased with increased management time. The complexity of soil bacterial and fungal microbial community relationships was higher in IM forests than in EM forests under the same management time, which may be attributed to the fact that continuous nutrient addition in IM forests selects some special microbial taxa, resulting in the proliferation of certain specific microbes to increase the efficiency of the conversion and decomposition of compounds in the soil (Wan et al. 2021; Zhang et al. 2023). It has been found that the abundance of functional genes of denitrifying bacteria in IM moso bamboo forests is higher than in EM forests, which can promote soil N cycling (Zheng et al. 2022). This study quantified the deterministic and stochastic processes of microbial community assembly based on a null model. The construction of soil microbial communities in *C. cathayensis* forests was found to be dominated by stochastic processes regardless of the changes in management methods and management time. Consistent with the results of our Hypothesis 3, with the increase of management time, the stochasticity decreased, and the determinism increased in the construction process of soil bacterial communities in IM forests, while the determinism gradually reduced and the stochasticity increased in EM forests. This result may come from nutrient selection, which increases the certainty of the soil bacterial community during the construction process (Wang, Zhang, et al. 2025). Research indicates that in disturbed soil ecosystems, stochastic factors primarily govern microbial community assembly processes (Liu et al. 2021). Among these processes, homogenizing dispersal and dispersal limitation predominantly shaped bacterial communities, whereas fungal communities were mainly influenced by drift (Cabrera‐Hernandez et al. 2025). In addition, soil pH is widely recognized as a key regulator of both stochastic and deterministic assembly processes in soil microbial communities (Tripathi et al. 2018). Under acidic soil conditions (pH ≤ 5.5), microbial assembly processes were more stochastic and driven primarily by homogenizing dispersal, whereas under neutral conditions (pH 5.5–8.5), deterministic processes, especially variable selection, dominated (Huang et al. 2025). In this study, soil pH decreased significantly with increasing management time, especially more pronounced in IM forests, which may be another key driver of the increase in deterministic processes in soil bacterial communities under IM forests. Some studies have found that soil pH and SOM content can affect bacterial community assembly processes; for example, pH in acidic soil may exert selective pressure on soil microbial survival (Feng et al. 2018; Zhang et al. 2023).

### 4.4 Limitations and Future Prospectives

While this study provides valuable insights into the effects of forest management methods and durations on soil microbial communities in *C. cathayensis* forests, several limitations should be acknowledged. First, the interpretation of microbial functional genes remains insufficient, as the study primarily focused on taxonomic composition and diversity. Understanding the functional potential of microbial communities, particularly genes involved in key metabolic processes such as C, N, and P cycling, would deepen our mechanistic understanding of how management practices influence soil ecosystem functioning. Future research should employ metagenomic sequencing to explore the expression and regulation of microbial functional genes under different management regimes. This approach could reveal how specific management practices alter microbial metabolic pathways, nutrient cycling, and ecosystem resilience. Secondly, it is necessary to expand the number of tree species. The current research is mainly applicable to guiding the sustainable management of *C. cathayensis*. Furthermore, how other soil microorganisms (e.g., nematodes) respond to changes in forest management methods and time is also worthy of attention. Future studies can address these gaps and bridge the knowledge gap between microbial community structure and function, offering practical guidance for balancing productivity and ecological sustainability in managed forest ecosystems.

## 5 Conclusion

Unlike the different management methods, sustained forest management significantly affected the diversity of soil microbial species in *C. cathayensis* forest. The soil microbial species diversity decreased to a lower level after 15 years of management and maintained a relatively stable state thereafter. The interspecies relationships between soil bacterial and fungal communities under different management methods and management times were mainly positive synergistic, and the microbial interspecies relationships in IM forests were more complex than those in EM forests. The main drivers of the construction of soil bacterial and fungal communities were stable in the time series under both management methods, and stochastic processes mainly controlled the construction of bacterial and fungal communities. The current study results suggest that short‐term intensive management positively affects forest soil microbial diversity, community structure, and network complexity.

## Author Contributions

**Cheng Huang:** conceptualization (equal), formal analysis (equal), investigation (equal), methodology (equal), software (equal), writing – original draft (equal). **Hua Liu:** funding acquisition (equal), methodology (equal), project administration (equal), resources (equal), writing – review and editing (equal). **Shu‐Yi‐Dan Zhou:** methodology (equal), software (equal), writing – review and editing (equal). **Linyun Mou:** writing – review and editing (equal). **Lingjun Cui:** writing – review and editing (equal). **Lan Yao:** writing – review and editing (equal). **Yuhua Ma:** investigation (equal), writing – review and editing (equal). **Fasih Ullah Haider:** writing – review and editing (equal). **Songling Fu:** funding acquisition (equal), project administration (equal), resources (equal), supervision (equal), writing – review and editing (equal). **Xu Li:** conceptualization (equal), supervision (equal), writing – review and editing (equal).

## Conflicts of Interest

The authors declare no conflicts of interest.

## Supporting information


**Data S1:** ece372173‐sup‐0001‐supinfo.docx.

**Data S2:** ece372173‐sup‐0002‐supinfo.xlsx.

## Data Availability

All raw data generated in this study have been uploaded to the NCBI BioProject database under accession number (PRJNA1298008). All the data analysis results obtained during this study are included in the manuscript and its Supporting Information—S1.
