# Supplementary material for: Management Methods and Duration Induces Changes in Soil Microbial Communities of Carya cathayensis var. dabeishansis Forests
Source: Ecol Evol. 2025 Sep 14;15(9):e72173. doi: 10.1002/ece3.72173 (PMC12434178; doi:10.1002/ece3.72173)
Supplement: Supplementary file 1 — Data S1: ece372173‐sup‐0001‐supinfo.docx. [file ECE3-15-e72173-s002.docx]

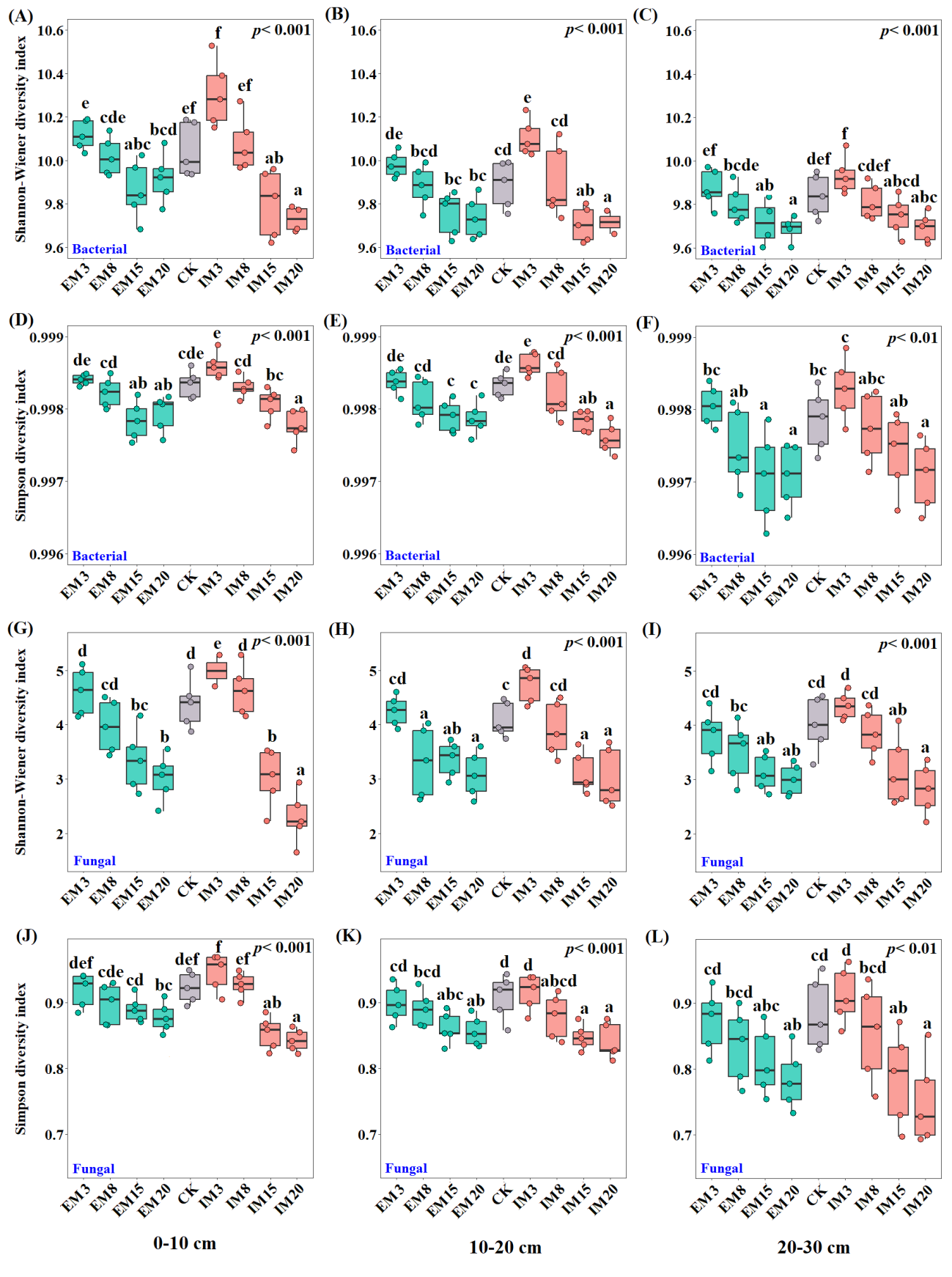


**Figure S1 Changes in the α diversity of soil microorganism communities in each soil layer under the different management methods and durations. Note: *, *p* < 0.05; **, *p* < 0.01; ***, *p* < 0.001.**


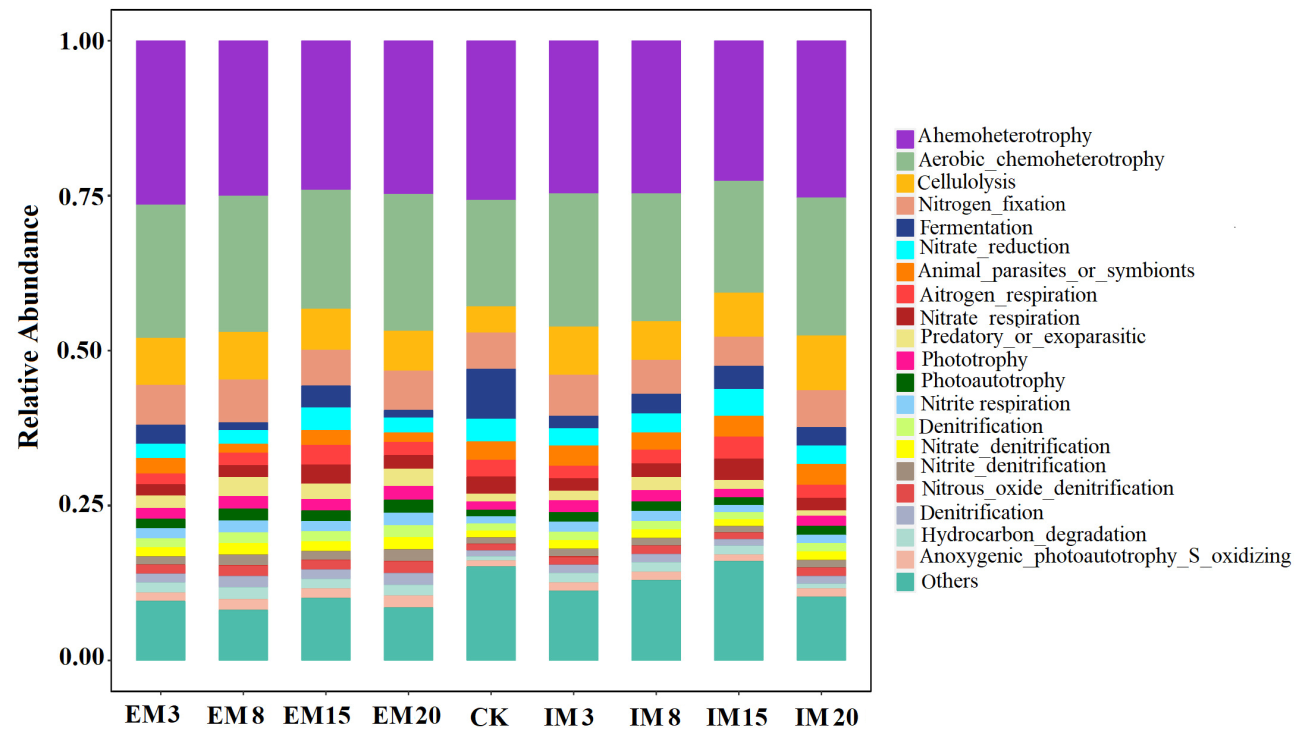


**Figure S2 The main ecological functions of soil** **bacterial communities annotated from FAPROTAX database**


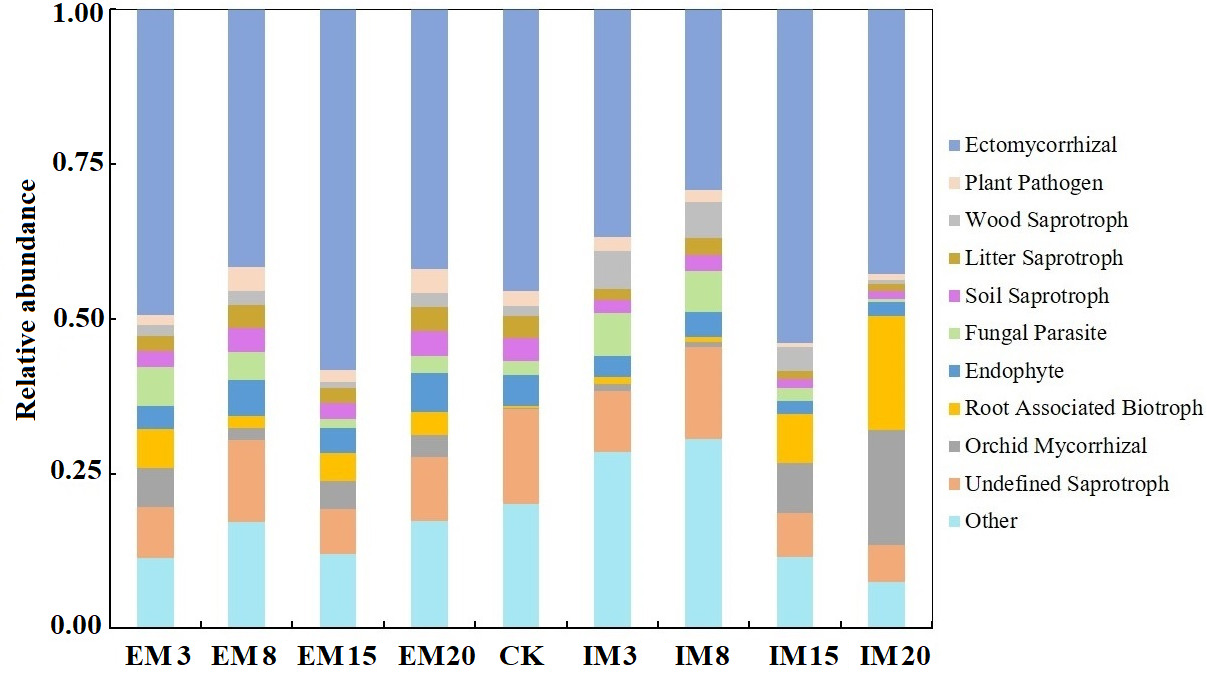


**Figure S3 Prediction of ecological function of soil** **fungal community based on FUNGuild.**

**Table. 1 Basic information of the plot.**

| Stand characteristics | CK | EM3 | EM8 | EM15 | EM20 | IM3 | IM8 | IM15 | IM20 |
| --- | --- | --- | --- | --- | --- | --- | --- | --- | --- |
| Location | 115°51´02" E 31°15´12 "N | 115°50´31" E 31°15´20 "N | 115°50´26"E  31°15´23"N | 115°50´58"E  31°15´13"N | 115°50´52"E  31°15´19"N | 115°22´51"E  31°28´04"N | 115°22´54"E  31°28´15"N | 115°22´47"E  31°28´16"N | 115°22´53"E  31°29´41"N |
| Altitude (m) | 747 | 720 | 746 | 716 | 723 | 735 | 750 | 678 | 632 |
| Slope (°) | 24 | 25 | 20 | 27 | 20 | 25 | 28 | 30 | 30 |
| Mean tree DBH (cm) | 12.9±0.5^a^ | 18.8±0.9^bc^ | 20.4±1.2^cd^ | 17.9±1.1^bc^ | 20.5±0.8^cd^ | 16.8±0.9^b^ | 22.7±1.3^d^ | 18.8±0.7^bc^ | 22.9±0.8^d^ |
| Mean tree height (m) | 9.5±0.2^d^ | 12.1±0.7^d^ | 11.8±0.4^cd^ | 11.6±0.1^cd^ | 11.4±0.4^cd^ | 10.2±0.3^ab^ | 10.9±0.4^bc^ | 12.3±0.4^d^ | 14.32±0.3^e^ |
| Stand density (trees ha^-1^) | 1150.0±22.4^e^ | 765.0±70.4^d^ | 660.0±49.1^bcd^ | 1080.0±76.4^e^ | 725.0±82.5^cd^ | 795.0±111.4^d^ | 480.0±21.5^ab^ | 535.0±49.1^abc^ | 445.0±54.4^a^ |
| Stand basal area (m^2^ ha^-1^) | 20.5±1.7^abc^ | 26.4±1.9^bc^ | 23.7±2.4^bc^ | 34.6±4.8^d^ | 28.5±2.2^cd^ | 19.4±3.3^ab^ | 20.5±1.9^abc^ | 15.3±1.5^a^ | 19.1±1.5^ab^ |
| Tree species richness | 9.4±1.2^c^ | 4±0.8^b^ | 1±0.0^a^ | 2.2±0.5^a^ | 2.6±0.8^ab^ | 1.4±0.4^a^ | 1.0±0.0^a^ | 1.0±0.0^a^ | 1.0±0.0^a^ |
| Shrub layer cover (%) | 39.9±6.8^c^ | 10.1±20.2^b^ | 2.1±0.5^a^ | 1.8±0.6^a^ | 0.5±0.3^a^ | 1.2±0.4^a^ | 0.5±0.1^a^ | 0.2±0.1^a^ | 0.2±0.1^a^ |
| Herb layer cover (%) | 34.5±3.0^a^ | 76.1±6.0^bc^ | 56.4±4.8^b^ | 30.7±1.5^a^ | 31.9±4.5^a^ | 44.8±6.1^c^ | 38.6±6.8^a^ | 21.7±4.0^a^ | 28.0±4.0^a^ |

Note: Different lowercase letters indicate significant differences in forest stands.

**Table S2 Comparison of species composition of soil bacterial communities under the different management methods and durations.**

|  | 0-10 cm | | 10-20 cm | | 20-30 cm | |
| --- | --- | --- | --- | --- | --- | --- |
|  | *R^2^* | *p* | *R^2^* | *p* | *R^2^* | *p* |
| EM-20 vs EM-15 | 0.113 | 0.379 | 0.113 | 0.381 | 0.182 | 0.032 * |
| EM-20 vs EM-8 | 0.130 | 0.167 | 0.130 | 0.160 | 0.257 | 0.015 * |
| EM-20 vs EM-3 | 0.172 | 0.010* | 0.172 | 0.008** | 0.175 | 0.059 |
| EM-20 vs IM-20 | 0.283 | 0.011* | 0.283 | 0.010* | 0.106 | 0.465 |
| EM-20 vs IM-15 | 0.205 | 0.007** | 0.205 | 0.010* | 0.258 | 0.015 * |
| EM-20 vs IM-8 | 0.164 | 0.022* | 0.164 | 0.026* | 0.190 | 0.007 ** |
| EM-20 vs IM-3 | 0.162 | 0.020* | 0.162 | 0.015* | 0.222 | 0.008 ** |
| EM-20 vs CK | 0.140 | 0.045* | 0.140 | 0.041* | 0.185 | 0.024 * |
| EM-15 vs EM-8 | 0.124 | 0.236 | 0.124 | 0.249 | 0.129 | 0.140 |
| EM-15 vs EM-3 | 0.193 | 0.018* | 0.193 | 0.024* | 0.114 | 0.357 |
| EM-15 vs IM-20 | 0.360 | 0.012* | 0.360 | 0.007** | 0.219 | 0.008 ** |
| EM-15 vs IM-15 | 0.258 | 0.007** | 0.258 | 0.009** | 0.337 | 0.010 * |
| EM-15 vs IM-8 | 0.214 | 0.009** | 0.214 | 0.013* | 0.327 | 0.009 ** |
| EM-15 vs IM-3 | 0.197 | 0.008** | 0.197 | 0.009** | 0.302 | 0.009 ** |
| EM-15 vs CK | 0.152 | 0.048* | 0.152 | 0.070 | 0.141 | 0.094 |
| EM-8 vs EM-3 | 0.199 | 0.015* | 0.199 | 0.003** | 0.146 | 0.129 |
| EM-8 vs IM-20 | 0.359 | 0.006** | 0.359 | 0.009** | 0.273 | 0.012 * |
| EM-8 vs IM-15 | 0.230 | 0.010* | 0.230 | 0.007** | 0.369 | 0.007 ** |
| EM-8 vs IM-8 | 0.196 | 0.006** | 0.196 | 0.006** | 0.368 | 0.011 * |
| EM-8 vs IM-3 | 0.176 | 0.014* | 0.176 | 0.020* | 0.328 | 0.012 * |
| EM-8 vs CK | 0.171 | 0.024* | 0.171 | 0.011* | 0.156 | 0.061 |
| EM-3 vs IM-20 | 0.260 | 0.011* | 0.260 | 0.011* | 0.194 | 0.020 * |
| EM-3 vs IM-15 | 0.222 | 0.009** | 0.222 | 0.013* | 0.285 | 0.006 ** |
| EM-3 vs IM-8 | 0.191 | 0.026* | 0.191 | 0.019* | 0.281 | 0.010 * |
| EM-3 vs IM-3 | 0.175 | 0.007** | 0.175 | 0.008** | 0.254 | 0.009 ** |
| EM-3 vs CK | 0.168 | 0.019* | 0.168 | 0.018* | 0.131 | 0.240 |
| IM-20 vs IM-15 | 0.243 | 0.008** | 0.243 | 0.010* | 0.194 | 0.005 ** |
| IM-20 vs IM-8 | 0.249 | 0.014* | 0.249 | 0.014* | 0.120 | 0.261 |
| IM-20 vs IM-3 | 0.221 | 0.008** | 0.221 | 0.007** | 0.124 | 0.309 |
| IM-20 vs CK | 0.355 | 0.007** | 0.355 | 0.004** | 0.191 | 0.014 * |
| IM-15 vs IM-8 | 0.167 | 0.022* | 0.167 | 0.036* | 0.218 | 0.019 * |
| IM-15 vs IM-3 | 0.149 | 0.076 | 0.149 | 0.110 | 0.190 | 0.010 * |
| IM-15 vs CK | 0.237 | 0.006** | 0.237 | 0.014* | 0.280 | 0.008 ** |
| IM-8 vs IM-3 | 0.136 | 0.227 | 0.136 | 0.193 | 0.125 | 0.312 |
| IM-8 vs CK | 0.232 | 0.008** | 0.232 | 0.007** | 0.284 | 0.009 ** |
| IM-3 vs CK | 0.207 | 0.016* | 0.207 | 0.010* | 0.252 | 0.010 * |

Note: *, *p* < 0.05; **, *p* < 0.01; ***, *p* < 0.001. The same below.

**Table S3** **Comparison of species composition of soil fungal communities under the** **different management methods and durations (continued).**

|  | 0-10 cm | | 10-20 cm | | 20-30 cm | |
| --- | --- | --- | --- | --- | --- | --- |
|  | *R^2^* | *p* | *R^2^* | *p* | *R^2^* | *p* |
| EM-20 vs EM-15 | 0.133 | 0.025* | 0.176 | 0.012* | 0.122 | 0.109 |
| EM-20 vs EM-8 | 0.136 | 0.037 * | 0.278 | 0.009 ** | 0.110 | 0.475 |
| EM-20 vs EM-3 | 0.262 | 0.016 * | 0.253 | 0.010 * | 0.217 | 0.008 ** |
| EM-20 vs IM-20 | 0.201 | 0.006 ** | 0.266 | 0.011 * | 0.138 | 0.010 * |
| EM-20 vs IM-15 | 0.143 | 0.020 * | 0.172 | 0.022 * | 0.133 | 0.049 * |
| EM-20 vs IM-8 | 0.192 | 0.007** | 0.249 | 0.007 ** | 0.153 | 0.010* |
| EM-20 vs IM-3 | 0.243 | 0.010 * | 0.391 | 0.012 * | 0.276 | 0.007 ** |
| EM-20 vs CK | 0.258 | 0.013 * | 0.259 | 0.008 ** | 0.110 | 0.500 |
| EM-15 vs EM-8 | 0.134 | 0.010 * | 0.202 | 0.008 ** | 0.131 | 0.030 * |
| EM-15 vs EM-3 | 0.228 | 0.003** | 0.180 | 0.006 ** | 0.200 | 0.009 ** |
| EM-15 vs IM-20 | 0.186 | 0.012 * | 0.193 | 0.012 * | 0.117 | 0.193 |
| EM-15 vs IM-15 | 0.132 | 0.007** | 0.127 | 0.007 ** | 0.123 | 0.057 |
| EM-15 vs IM-8 | 0.182 | 0.011 * | 0.173 | 0.009 ** | 0.137 | 0.019 * |
| EM-15 vs IM-3 | 0.204 | 0.009** | 0.291 | 0.008 ** | 0.257 | 0.009 ** |
| EM-15 vs CK | 0.250 | 0.010 * | 0.205 | 0.008 ** | 0.150 | 0.015 * |
| EM-8 vs EM-3 | 0.250 | 0.011 * | 0.256 | 0.015 * | 0.171 | 0.009 ** |
| EM-8 vs IM-20 | 0.216 | 0.012 * | 0.315 | 0.006 ** | 0.137 | 0.075 |
| EM-8 vs IM-15 | 0.164 | 0.013 * | 0.232 | 0.010* | 0.154 | 0.007** |
| EM-8 vs IM-8 | 0.206 | 0.012* | 0.286 | 0.006 ** | 0.172 | 0.006 ** |
| EM-8 vs IM-3 | 0.230 | 0.007** | 0.442 | 0.008 ** | 0.259 | 0.009 ** |
| EM-8 vs CK | 0.242 | 0.011 * | 0.288 | 0.017 * | 0.121 | 0.216 |
| EM-3 vs IM-20 | 0.326 | 0.004 ** | 0.254 | 0.011* | 0.209 | 0.011 * |
| EM-3 vs IM-15 | 0.268 | 0.010 * | 0.183 | 0.009** | 0.216 | 0.013 * |
| EM-3 vs IM-8 | 0.311 | 0.005** | 0.222 | 0.008 ** | 0.243 | 0.004 ** |
| EM-3 vs IM-3 | 0.321 | 0.009 ** | 0.348 | 0.007** | 0.299 | 0.008 ** |
| EM-3 vs CK | 0.337 | 0.009 ** | 0.243 | 0.011 * | 0.213 | 0.010 * |
| IM-20 vs IM-15 | 0.172 | 0.011* | 0.186 | 0.008 ** | 0.125 | 0.016 * |
| IM-20 vs IM-8 | 0.239 | 0.010 * | 0.243 | 0.010 * | 0.148 | 0.008** |
| IM-20 vs IM-3 | 0.299 | 0.010* | 0.380 | 0.008 ** | 0.264 | 0.007** |
| IM-20 vs CK | 0.341 | 0.007** | 0.287 | 0.007 ** | 0.154 | 0.011 * |
| IM-15 vs IM-8 | 0.168 | 0.010 * | 0.149 | 0.011* | 0.128 | 0.160 |
| IM-15 vs IM-3 | 0.228 | 0.006 ** | 0.284 | 0.007 ** | 0.262 | 0.013 * |
| IM-15 vs CK | 0.268 | 0.006 ** | 0.206 | 0.006 ** | 0.166 | 0.014 * |
| IM-8 vs IM-3 | 0.260 | 0.012* | 0.300 | 0.011 * | 0.249 | 0.006 ** |
| IM-8 vs CK | 0.333 | 0.006 ** | 0.266 | 0.008 ** | 0.187 | 0.007 ** |
| IM-3 vs CK | 0.327 | 0.013 * | 0.395 | 0.016 * | 0.289 | 0.010 * |

**Table S4 Statistics of soil chemical, physical, and biological indicators in** ***C. cathayensis var. dabeishansis* forests under** **different management intensities and durations (0-10 cm).**

|  | CK | IM-3 | IM-8 | IM-15 | IM-20 | EM-3 | EM-8 | EM-15 | EM-20 |
| --- | --- | --- | --- | --- | --- | --- | --- | --- | --- |
| MC | 15.73±0.82abc | 17.55±0.75c | 13.61±0.75ab | 12.71±0.94a | 13.31±1.12a | 16.74±0.94bc | 15.53±1.04abc | 15.73±1.22abc | 14.49±1.04abc |
| EC | 63.28±4.96bc | 66.14±7.18bc | 68.16±2.55c | 74.40±2.69c | 71.22±2.57c | 54.34±2.16ab | 45.42±1.47a | 49.18±4.08a | 44.50±4.41a |
| BD | 1.18±0.01bc | 0.87±0.04a | 1.07±0.05b | 1.26±0.04cd | 1.35±0.07d | 1.16±0.04bc | 1.01±0.07ab | 1.11±0.03bc | 1.24±0.06cd |
| SP | 55.62±0.54bc | 67.09±1.63d | 59.77±1.79c | 52.30±1.68ab | 48.98±2.75a | 56.3±1.70bc | 61.74±2.61cd | 58.11±1.04bc | 53.06±2.45ab |
| pH | 5.31±0.03e | 5.19±0.06cd | 4.88±0.04a | 4.89±0.02a | 4.81±0.04a | 5.23±0.05de | 5.08±0.02bc | 5.02±0.03b | 5.11±0.03bc |
| TN | 2.33±0.15a | 4.15±0.40e | 3.37±0.15d | 2.91±0.12abcd | 2.99±0.10bcd | 3.18±0.14cd | 2.66±0.26abc | 2.47±0.10ab | 2.54±0.13ab |
| TP | 0.69±0.03bc | 0.70±0.06c | 0.60±0.03bc | 0.41±0.05a | 0.35±0.05a | 0.64±0.04bc | 0.57±0.04b | 0.41±0.04a | 0.33±0.03a |
| TK | 24.29±0.62bc | 27.11±1.47c | 25.02±1.33bc | 24.49±0.92bc | 24.44±0.88bc | 25.27±0.38bc | 22.07±0.54b | 18.09±1.49a | 17.41±0.62a |
| SOC | 27.61±1.62cd | 31.9±1.99d | 25.04±0.72bc | 21.22±0.76ab | 18.42±2.31a | 29.11±1.36cd | 24.52±1.93bc | 19.38±1.43a | 18.13±1.10a |
| AN | 264.45±13.18a | 406.32±32.25c | 330.18±12.58b | 300.02±9.39ab | 289.38±2.79ab | 293.49±13.11ab | 288.32±32.65ab | 268.35±10.11a | 258.03±10.18a |
| AP | 21.65±1.59d | 20.36±1.28cd | 15.16±0.55ab | 14.94±0.56ab | 13.59±0.51a | 18.78±1.39bcd | 17.11±1.69abc | 15.68±1.69ab | 15.95±1.53ab |
| AK | 70.61±4.17bcd | 93.9±7.19e | 79.66±2.64de | 75.93±2.43cd | 77.07±4.05cd | 58.25±2.38ab | 71.39±5.05bcd | 53.85±8.51a | 62.47±5.83abc |
| MBP | 7.30±0.09d | 7.24±0.35d | 5.76±0.16ab | 5.79±0.21ab | 5.11±0.16a | 7.11±0.20cd | 6.43±0.51bc | 6.09±0.30b | 5.78±0.18ab |
| MBC | 184.87±11.99abc | 280.10±15.67e | 245.55±17.95de | 160.67±5.91ab | 152.95±10.88a | 283.49±10.19e | 210.38±28.04cd | 205.63±9.67bcd | 174.44±11.37abc |
| MBN | 82.97±1.62bc | 87.05±3.94c | 85.97±5.81c | 74.37±2.57ab | 68.34±0.69a | 85.73±6.91c | 73.09±3.53ab | 70.45±1.98a | 70.55±0.86a |
| ACP | 59.64±2.33a | 70.28±2.29b | 65.02±1.68ab | 63.05±1.75a | 62.55±2.23a | 61.25±2.55a | 59.75±3.09a | 57.64±2.86a | 58.11±1.88a |
| UE | 16.65±0.57bc | 20.00±0.74a | 17.34±1.06c | 15.51±0.32abc | 16.94±0.56c | 17.61±0.38c | 14.35±0.60ab | 13.47±0.99a | 13.74±1.19a |
| SC | 2.47±0.09cd | 2.81±0.08e | 2.24±0.11bc | 1.92±0.07a | 1.82±0.06a | 2.9±0.12e | 2.54±0.08d | 2.02±0.07ab | 2.01±0.05ab |
| BG | 60.64±2.33abc | 65.85±1.79c | 63.78±2.57c | 55.21±3.66ab | 54.72±2.74a | 64.51±2.22c | 63.26±1.89bc | 57.89±3.59abc | 57.56±0.69abc |
| Pro | 9.49±0.49abc | 10.97±0.60c | 9.78±0.72bc | 9.93±0.43bc | 9.04±0.23ab | 9.63±0.36abc | 8.79±0.34ab | 8.94±0.69ab | 8.15±0.36a |

Note: Values are the mean ± standard error; There are significant differences in the one-way ANOVA of various compound patterns with different letters (LSD, *p* < 0.05).MC, soil water content; EC, conductivity value; BD, bulk density; SP, porosity of soil; TN, total nitrogen; TP, total phosphorus; TK, total potassium; SOC, soil organic carbon; AN, alkali hydrolyzed nitrogen; AP, available phosphorus; AK, available potassium; MBP, soil microbial biomass phosphorus; MBC, soil microbial biomass carbon; MBN, soil microbial biomass nitrogen; ACP, acid phosphatase; UE, urease; SC, sucrase; BG, β-1,4-glucosidase; Pro, protease. The same applies below.

**Table S5 Statistics of soil chemical, physical, and biological indicators in *C. cathayensis var. dabeishansis* forests under different management intensities and durations (10-20 cm).**

|  | CK | IM-3 | IM-8 | IM-15 | IM-20 | EM-3 | EM-8 | EM-15 | EM-20 |
| --- | --- | --- | --- | --- | --- | --- | --- | --- | --- |
| MC | 13.02±0.64a | 16.42±1.01a | 14.23±1.17a | 13.31±0.89a | 13.71±1.56a | 15.68±0.67a | 14.47±0.95a | 13.89±1.17a | 14.03±0.98a |
| EC | 34.93±2.03a | 52.92±1.67b | 56.50±3.29b | 55.52±3.89b | 51.42±2.88b | 40.53±1.35a | 37.80±3.33a | 34.72±2.81a | 34.62±3.91a |
| BD | 1.15±0.06ab | 1.09±0.03a | 1.30±0.06cd | 1.46±0.06e | 1.47±0.07e | 1.19±0.03abc | 1.18±0.03abc | 1.26±0.02bcd | 1.36±0.06de |
| SP | 56.66±2.11de | 58.89±1.15e | 50.81±2.14bc | 44.90±2.12a | 44.57±2.59a | 55.05±1.09cde | 55.57±1.23cde | 52.30±0.65bcd | 48.50±2.19ab |
| pH | 5.21±0.05d | 5.18±0.06bcd | 5.02±0.06abc | 5.01±0.06ab | 4.92±0.04a | 5.20±0.09cd | 5.14±0.04bcd | 5.14±0.04bcd | 5.14±0.03bcd |
| TN | 1.67±0.26a | 3.01±0.28d | 2.73±0.27cd | 2.34±0.17bc | 2.1±0.17ab | 2.18±0.14abc | 1.93±0.18ab | 1.94±0.11ab | 1.79±0.08ab |
| TP | 0.41±0.06bc | 0.53±0.06d | 0.43±0.06cd | 0.32±0.03abc | 0.26±0.02a | 0.34±0.04abc | 0.30±0.03abc | 0.29±0.01ab | 0.28±0.03a |
| TK | 22.16±0.62cd | 22.37±0.83cd | 22.1±1.63cd | 23.14±0.87d | 20.35±0.40bcd | 17.54±0.51ab | 17.58±0.65ab | 19.67±1.63abc | 17.07±0.80a |
| SOC | 18.19±1.12b | 18.14±1.90b | 16.12±0.99ab | 17.00±1.61ab | 12.90±1.16a | 16.9±0.98ab | 15.75±1.71ab | 14.77±1.26ab | 14.29±2.05ab |
| AN | 205.81±13.44a | 285.99±19.29c | 262.92±21.61bc | 206.39±17.42a | 180.16±20.08a | 218.48±9.04ab | 212.03±18.25a | 190.98±5.38a | 181.17±11.22a |
| AP | 12.27±0.51d | 10.75±0.45c | 8.03±0.59ab | 7.81±0.38ab | 7.01±0.51a | 10.52±0.47c | 8.95±0.51b | 7.91±0.43ab | 7.49±0.38ab |
| AK | 52.07±2.40abc | 67.32±1.98d | 54.84±1.58c | 55.02±2.44c | 53.04±3.87bc | 50.06±1.87abc | 42.45±2.97a | 43.64±2.26ab | 47.81±6.18abc |
| MBP | 5.70±0.16ab | 6.58±0.53b | 6.05±0.69ab | 5.55±0.29ab | 5.45±0.14ab | 6.31±0.23ab | 6.28±0.31ab | 5.44±0.38ab | 5.16±0.33a |
| MBC | 189.81±11.96bcd | 209.83±15.13d | 160.76±17.15abc | 159.98±11.48abc | 137.35±13.63a | 200.25±14.13cd | 206.85±21.86d | 156.87±3.69abc | 145.78±7.81ab |
| MBN | 72.05±2.55cd | 74.05±2.66d | 70.11±3.92bcd | 64.25±1.40abc | 62.73±1.69ab | 70.86±3.83cd | 64.55±2.13abc | 61.13±0.77a | 58.96±0.95a |
| ACP | 43.76±1.34a | 44.97±2.37a | 42.69±3.43a | 41.62±2.45a | 43.52±2.87a | 45.91±1.00a | 43.87±3.87a | 40.76±2.20a | 40.05±2.65a |
| UE | 14.08±1.01b | 16.74±0.28c | 16.58±0.55c | 14.41±0.57bc | 14.81±0.73bc | 14.81±0.24bc | 13.17±0.88ab | 12.82±0.41ab | 11.62±1.35a |
| SC | 1.89±0.14a | 2.38±0.06b | 2.06±0.08ab | 2.10±0.06ab | 1.93±0.07a | 2.32±0.13b | 2.38±0.18b | 1.94±0.11a | 2.10±0.03ab |
| BG | 54.76±4.75a | 56.53±2.13a | 55.15±2.01a | 56.68±1.57a | 48.23±3.22a | 56.24±2.06a | 54.30±1.97a | 50.27±3.81a | 52.12±2.53a |
| Pro | 6.69±0.42a | 9.19±0.32de | 8.68±0.54e | 8.25±0.54bcde | 8.22±0.31bcde | 8.41±0.42cde | 7.02±0.53abc | 7.35±0.60abcd | 6.8±0.50ab |

**Table S6 Statistics of soil chemical, physical, and biological indicators in *C. cathayensis var. dabeishansis* forests under different management intensities and durations (20-30 cm).**

|  | CK | IM-3 | IM-8 | IM-15 | IM-20 | EM-3 | EM-8 | EM-15 | EM-20 |
| --- | --- | --- | --- | --- | --- | --- | --- | --- | --- |
| MC | 14.9±0.59ab | 17.36±1.24c | 16.14±1.63bc | 12.52±0.84a | 12.63±0.66a | 15.86±1.08bc | 13.59±0.91ab | 13.13±0.53ab | 12.21±1.08a |
| EC | 32.30±2.99ab | 49.20±3.90c | 49.32±3.75c | 50.62±2.36c | 51.68±2.60c | 38.77±2.75b | 26.89±2.06a | 26.84±2.32a | 28.02±2.21a |
| BD | 1.35±0.03ab | 1.31±0.04ab | 1.34±0.07ab | 1.46±0.06b | 1.43±0.07b | 1.20±0.06a | 1.30±0.04ab | 1.35±0.05ab | 1.41±0.05b |
| SP | 49.01±1.11ab | 50.56±1.44ab | 49.47±2.65ab | 44.94±2.28a | 46.01±2.66a | 54.71±2.09b | 50.87±1.68ab | 49.21±1.88ab | 46.88±1.99a |
| pH | 5.35±0.06b | 5.22±0.12ab | 5.14±0.05ab | 5.05±0.09a | 5.16±0.07ab | 5.33±0.02b | 5.23±0.04ab | 5.22±0.06ab | 5.21±0.04ab |
| TN | 1.60±0.22a | 2.50±0.21c | 2.14±0.32bc | 1.88±0.11ab | 1.61±0.07a | 1.89±0.11ab | 1.50±0.08a | 1.64±0.11ab | 1.48±0.07a |
| TP | 0.54±0.01de | 0.57±0.04e | 0.48±0.04cde | 0.36±0.04ab | 0.26±0.02a | 0.54±0.04de | 0.40±0.04bcd | 0.37±0.04abc | 0.33±0.03ab |
| TK | 23.07±0.86a | 23.27±1.58a | 22.10±2.41a | 22.28±0.93a | 21.21±1.80a | 19.70±1.63a | 17.87±0.55a | 18.03±1.64a | 18.12±2.00a |
| SOC | 14.94±1.20c | 15.68±2.13c | 13.65±0.76bc | 13.46±1.08bc | 9.36±1.03a | 14.13±0.63bc | 12.36±0.99abc | 12.02±1.20abc | 10.45±0.91ab |
| AN | 150.74±5.82a | 236.69±22.36c | 208.21±30.60bc | 175.83±9.20ab | 154.11±11.16a | 182.91±18.04ab | 149.80+12.41a | 162.48±3.76ab | 148.43±7.19a |
| AP | 13.82±1.13c | 10.07±1.40b | 6.29±0.69a | 6.51±0.95a | 6.13±0.81a | 7.85±0.88ab | 8.31±0.68ab | 6.83±0.74a | 8.99±0.67ab |
| AK | 49.46±2.65bc | 64.43±4.75d | 46.23±2.81abc | 46.84±2.95abc | 44.63±1.78abc | 51.24±4.30c | 41.46±4.37abc | 39.36±2.61ab | 37.31±2.52a |
| MBP | 5.69±0.27d | 5.42±0.28cd | 4.71±0.29abc | 4.82±0.11abc | 4.13±0.28a | 5.29±0.15cd | 4.94±0.15bc | 4.93±0.18bc | 4.50±0.15ab |
| MBC | 173.71±6.28b | 166.93±11.90b | 133.51±10.97ab | 141.12±9.22ab | 116.93±7.01a | 165.92±23.13b | 119.84±11.65a | 133.89±12.33ab | 122.31±11.77a |
| MBN | 51.64±1.44c | 53.06±0.96c | 50.18±1.16bc | 50.51±0.72bc | 45.91±0.93a | 49.68±1.55bc | 47.92±0.56ab | 46.29±1.37a | 45.42±0.66a |
| ACP | 42.52±2.42ab | 45.47±2.17ab | 43.39±1.47ab | 39.10±1.82a | 41.32±1.79ab | 46.50±1.91b | 44.12±1.72ab | 43.65±1.66ab | 42.72±3.87ab |
| UE | 12.10±0.42ab | 13.69±0.31b | 13.45±0.41b | 13.3±0.75b | 13.11±0.80b | 12.33±0.67ab | 12.79±0.43ab | 12.76±0.28ab | 11.33±0.45a |
| SC | 2.17±0.08c | 2.05±0.04bc | 1.80±0.06a | 1.83±0.09a | 1.83±0.05a | 1.98±0.06abc | 1.92±0.06ab | 1.87±0.09ab | 1.84±0.04a |
| BG | 53.01±3.92a | 50.84±3.45a | 47.25±1.85a | 49.65±1.97a | 47.14±2.32a | 47.30±3.63a | 47.07±1.84a | 44.14±2.23a | 44.05±2.35a |
| Pro | 6.19±0.35ab | 7.32±0.23cd | 7.16±0.32bcd | 7.42±0.22d | 6.76±0.16abcd | 6.64±0.25abcd | 6.37±0.24abc | 6.21±0.51ab | 5.93±0.34a |
